# Effect of Dietary Bioactive Compounds on Mitochondrial and Metabolic Flexibility

**DOI:** 10.3390/diseases4010014

**Published:** 2016-03-10

**Authors:** Jose C. E. Serrano, Anna Cassanye, Meritxell Martín-Gari, Ana Belen Granado-Serrano, Manuel Portero-Otín

**Affiliations:** Department of Experimental Medicine, University of Lleida, Av. Alcalde Rovira Roure 80, Lleida 25198, Spain; annakasa4@gmail.com (A.C.); meri_martin@medicina.udl.cat (M.M.-G.); anabgs@mex.udl.cat (A.B.G.-S.); manuel.portero@mex.udl.cat (M.P.-O.)

**Keywords:** metabolism, mitochondria, polyphenols, ω-3, fiber, insulin resistance

## Abstract

Metabolic flexibility is the capacity of an organism to adequately respond to changes in the environment, such as nutritional input, energetic demand, *etc.* An important player in the capacity of adaptation through different stages of metabolic demands is the mitochondrion. In this context, mitochondrial dysfunction has been attributed to be the onset and center of many chronic diseases, which are denoted by an inability to adapt fuel preferences and induce mitochondrial morphological changes to respond to metabolic demands, such as mitochondrial number, structure and function. Several nutritional interventions have shown the capacity to induce changes in mitochondrial biogenesis/degradation, oxidative phosphorylation efficiency, mitochondrial membrane composition, electron transfer chain capacity, *etc.*, in metabolic inflexibility states that may open new target options and mechanisms of action of bioactive compounds for the treatment of metabolic diseases. This review is focused in three well-recognized food bioactive compounds that modulate insulin sensitivity, polyphenols, ω-3 fatty acids and dietary fiber, by several mechanism of action, like caloric restriction properties and inflammatory environment modulation, both closely related to mitochondrial function and dynamics.

## 1. Introduction

Life is the condition that distinguishes physical entities from inorganic matter, having processes, such as the capacity for growth, reproduction, functional activity and continual change preceding death. During all of these processes, life is regulated by biochemical reactions, named metabolism, whereas with good environmental factors, it may be the adoption of the best metabolic pathway for the achievement of the next lifetime step or induce a maintenance stage waiting for better environmental conditions. At the cellular level, energy availability regulates most cellular functions. Energy sensors, for example the AMP-activated protein kinase-α (AMPKα), activated by falling cellular energy levels, acts to restore energy homoeostasis by switching on catabolic pathways that generate ATP, while switching off anabolic pathways and other processes consuming ATP [1]. In this sense, energy availability will predispose to cell survival, whereas energy unavailability will induce a static stage and in the worst situation, if it continuous with time, cell death. Thus, metabolic flexibility, the capacity of an organism, an organ or a single cell to adapt fuel oxidation to fuel availability to meet cell energy demands, is an important feature in the maintenance of life [2]. However, due to evolutionarily-acquired knowledge, the most common environmental condition in nature is the one where energy availability is scarce, and most living organisms do not have the capacity to induce metabolism adaptations in response to a continuous and prolonged energy availability stage. Translating this basic metabolism concept to the current human condition, most of the actual chronic degenerative diseases have as a common factor the inability to adapt to an excess of energy availability. For example, insulin resistance, the key defect in type 2 diabetes, is associated with a low capacity to adapt fuel oxidation to fuel availability [3]. This results in lower glucose oxidation during insulin-stimulated conditions and in a relative low activation of lipid catabolism, which further supports the accumulation of ectopic fat and lipotoxicity with a deleterious effect on insulin signaling.

**Figure 1:**
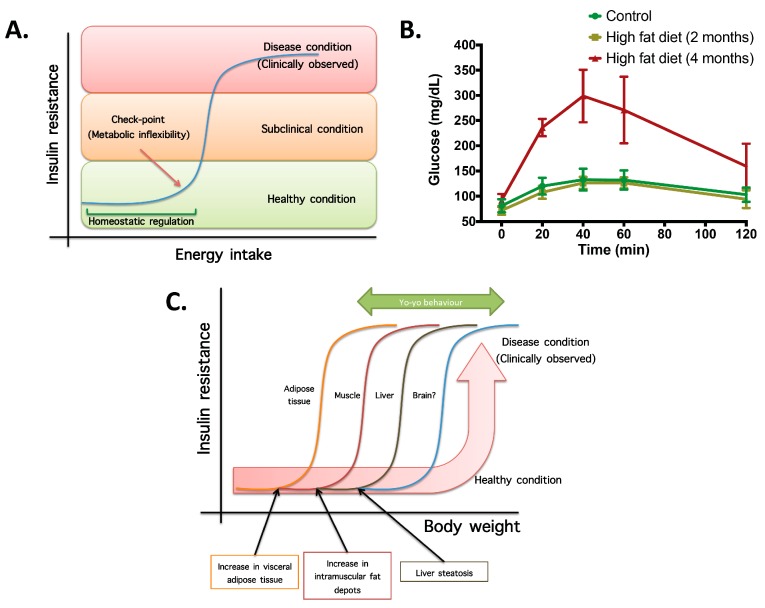
The concept of metabolic inflexibility and disease related outcomes. (**A**) Scheme of high-energy intake homeostatic regulation until a checkpoint, where energy storage systems are overloaded and subclinical and pathological conditions are observed. (**B**) Plasma glucose levels during a sub-cutaneous glucose tolerance test (2 g glucose/kg body weight) in controls and mice fed with a high-fat diet (34%) for two and four months. Insulin resistance, measured by means of blood glucose, is observed at four months of high-fat intake, whereas at two months, homeostatic compensation is still observed (unpublished observations). (**C**) Scheme of metabolic flexibility checkpoints in different organs for which, after passing organ-specific conditions, pathological features are observed. This explains the rapid change in metabolic flexibility after a weight reduction program and the yo-yo effect if the weight reduction is not maintained.

From a nutritional point of view, knowing that human metabolism is not well adapted to prolonged energy availability, an increase in energy expenditure or reductions of energy intake have been described as the main treatment objectives. However, most weight-loss programs for obese patients are known to usually show low percentages of success in terms of weight loss and, most importantly, in weight maintenance. This could be explained by several reasons:

(1) Not all organs and systems respond in a similar way to an increase or decrease in energy availability. Differences are observed in insulin sensitivity in different organs during the development from an insulin resistance stage to a clinically type 2 diabetes stage. It seems that the onset of type 2 diabetes or other clinical complications are a consequence of the increase in the number of organs with reduced insulin sensitivity. In the clinical setting, it is common to observe a rapid shift from a non-pathological (subclinical) to a disease condition (Figure 1A) that is triggered after exceeding a metabolic checkpoint where the organism is unable to maintain homeostasis. For example, Figure 1B shows that the intake of a high-fat diet in mice is well regulated after two months, until a possible accumulation of factors that triggers a disease condition observed at Month 4 with a clinically-observed feature (unpublished observations); suggesting that, at a certain point, the homeostatic compensation is overwhelmed, and pathological conditions could be observed. Even though, it is interesting to note in the same Figure 1B that at four months of a high-fat diet, the basal glucose levels are the same, although the organism is unable to maintain normal glycemia levels; implying that, in stress conditions (glucose overload), the capacity of the system to rapidly maintain homeostasis is compromised; albeit, finally, a homeostatic condition is reached (fasting state).
(2) In the same way, in the clinical setting, the effects of a mild reduction in body weight induce a rapid switch from a disease to a clinically non-pathological condition (Figure 1C). However, in this respect, frailty from this condition is also observed if an increase in body weight is observed. This implies that although most disease biomarkers are normalized during weight reduction programs, the metabolic system is unstable and liable to return to a disease condition. In other words, some organs still have metabolic inflexibility, explaining, in this way, the deleterious effects of “yo-yo” dieting.
(3) The total recovery of the metabolic inflexibility condition will imply the normalization of metabolic flexibility in all organs. Thus, combined treatments with multiple mechanisms of action are required for a better handling of metabolic diseases.

To understand disease progression, it is important to know what a healthy metabolic capacity constitutes. Individuals without metabolic diseases have the capacity to oxidize both carbohydrates and lipid-based fuels and to transition between these substrates in response to hormones and substrate signals. Thus, functional cellular defects in the energy transforming systems are likely to have profound systemic effects on overall metabolic health. Mitochondrial deficiencies limit the capacity of oxidative tissues to adapt fat oxidation to fat availability, leading to lipid accumulation in non-adipose tissue. These lipids interfere with the insulin signaling pathway and represent one of the key aspects that could contribute to metabolic imbalance [3].

There is increasing evidence for the concept of redox-driven alterations of mitochondrial function, as well as mitochondrial reactive oxygen species generation as a key event for the development of metabolic inflexibility. Diverse mitochondrial parameters vary between insulin-resistant and insulin-sensitive subjects, such as mitochondrial number and structure [4]. However, it is also important to note that the differences in metabolic imbalances could not be described solely in terms of mitochondrial quantity, rather by mitochondrial function. Many cells operate at basal levels that only require a part of their total bioenergetics capability. The differences between ATP produced by oxidative phosphorylation at basal and at maximal activity is termed reserve respiratory capacity [5]. Therefore, metabolic inflexibility limits the mitochondria from reaching their maximal respiratory capacity, because of several factors that predispose to an excess of energy storage.

## 2. Mitochondria Dynamic Regulation

**Figure 2:**
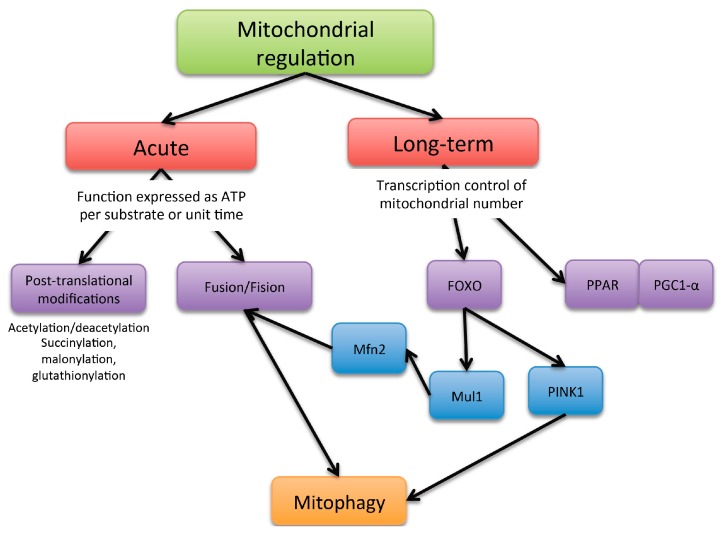
Scheme of the mechanism of mitochondria functioning regulation.

Mitochondria activity can be controlled through at least two major mechanism: acute ones, aimed at qualitatively modifying mitochondrial function, and longer-term transcriptional mechanisms, aimed at increasing mitochondrial number (Figure 2).

Acute mechanisms affect the intrinsic ability of mitochondria to generate ATP per molecule of nutrient or per unit of time. The energy requirements fluctuate constantly, and the metabolism of ATP is regulated to avoid futile energy expenditure and to cater to the specific needs of different tissues. Short-term regulators of oxidative phosphorylation exert their control in response to sudden changes of ATP demand.

The first level of regulation could be considered to occur by metabolic intermediates and substrate availability. As described by Chance and Williams in the 1950s, the metabolic states of mitochondria depend on substrate, oxygen and ADP levels [6]. The resting state of the mitochondria, defined as State 4, is where NADH is at 99% in the reduced form, and ADP is the rate limiting substance. If ADP is introduced into the system, ATP synthesis will be stimulated; O_2_ consumption will increase; and the rate limit will be determined by the activity of the respiratory chain; this last one is defined as State 3 or the “active state”. Other metabolism by-products, like ceramides, precursors for the predominant sphingolipids in the cell, inhibit mitochondrial complex III activity [7] and may be the cause the disruption of insulin sensitivity.

The electron transfer chain (ETC) has been demonstrated to be allosterically inhibited by ATP, whereby a futile overproduction of ATP is avoided [8]. Additionally, the catalytic activity of complex IV is regulated by the mitochondrial electric membrane potential [9], avoiding a hyperpolarization of the mitochondrial membrane and, thereby, avoiding excessive ROS production. Nitric oxide has also been demonstrated to regulate mitochondrial function at the level of cytochrome c oxidase by competing for the oxygen binding site of the enzyme [10].

In the same sense, post-translational modifications of ETC and ATP synthase have been demonstrated in several complexes’ catalytic activity. In fact, complex I and ATP synthase have been demonstrated to contain phosphorylation sites [11]. Additionally, other post-translational modifications, like protein acetylation, succinylation, glutathionylation and malonylation, have been described. Protein acetylation is a reversible mechanism linking the metabolic state and mitochondrial function. Upon fasting and high-fat feeding, the acetylation pattern of mitochondrial proteins strongly changes. For example, one week of high-fat diet enhanced silent mating type information regulation 2 homolog 3 (SIRT3) levels and prevented mitochondrial hyperacetylation [12], possibly to cope with the need to use fatty acid oxidation as the main path for energy production. Conversely, a prolonged high-fat diet decreased Sirt3 expression with the appearance of mitochondrial hyperacetylation and mitochondrial dysfunction, which suggests that, passing a homeostatic regulation capability threshold, dysfunction starts to emerge. 

Acute changes in mitochondria architecture have also been described, namely mitochondrial dynamics, where this involves fusion and fission processes. Cells under a well-fed condition maintain their mitochondria in a separated or fragmented state; while under fasting conditions, they tend to be in a fused state [13]. The main described regulators of the mitochondrial architecture for promoting fusion are mitofusin 1 (Mfn1), mitofusin 2 (Mfn2) and optic atrophy 1 (Opa1) proteins. Mitochondrial fission is regulated by mitochondrial fission factor (Mff) and dynamin-related protein 1 (Drp1). Changes in mitochondrial architecture addressed by fragmentation or fission processes may induce modifications in respiratory complex assembly, and the transition between fragmented and fused states allows mitochondria to reorganize and dispose of damaged elements through mitophagy. Notably, if the mitochondrial life cycle is attenuated, the accumulation of dysfunctional mitochondria will lead to an increase in oxidative stress that will lead to an increase of damaged mitochondria, entering a vicious circle.

Long-term regulators of oxidative phosphorylation could be considered as effectors involved in the regulation of mitochondrial biogenesis. Although mitochondrial biogenesis is regulated by a large number of coactivators and transcription factors, the proliferator-activated receptor coactivator-1α (PGC-1α) plays a role as a master regulator of such processes, upregulating the activity of nuclear respiratory factor 1 (NRF-1) and transcription factor A mitochondrial (TFAM); the two latter are key for mitochondrial DNA (mtDNA) replication and transcription. PGC-1α activity is highly regulated by post-transcriptional phosphorylation and acetylation. Reversible acetylation of PGC-1α significantly modifies its transcriptional activity, and it seems to be primarily controlled by silent mating type information regulation 2 homolog 1 (SIRT1), a nicotinamide adenine dinucleotide-dependent deacetylase and AMPKα activated with high levels of AMP in relation to ATP [14].

Long-term regulators can also permanently change the properties of the mitochondrial respiration, allowing mitochondrial differentiation, making them specialized for different cells and tissues. For example, specific activity capacity in certain complexes has been observed in tissues with diverse metabolic demands, which could be explained by the tissue-specific expression of ETC complexes’ isoforms. For example, complex IV isolated from cow lung has shown a 2.5-fold increased activity compared to liver complex IV [15].

In addition to defining mitochondrial dynamics, it is important to give an overview about the possible mechanism by which impairments in mitochondrial oxidative metabolism are observed. The decrease in substrate oxidation may affect electron flow through the ETC, causing electron leakage towards oxygen and the formation of superoxide. Superoxide and other ROS may damage several mitochondrial and cellular components and potentially result in either mitophagy (removal of damaged mitochondria) or, under high stress levels, apoptosis. The removal of mitochondria through mitophagy or the accumulation of damaged mitochondria in the case where mitophagy is suppressed could reduce the number of functioning mitochondria, resulting in decreased substrate oxidation. The reduced oxidation of fuels, particularly fatty acids, results in lipid accumulation, including deposition of metabolically-active lipid mediators, such as diacylglycerols and ceramides. Both have been shown to inhibit insulin signaling, inducing an insulin resistance state that may affect other organs.

However, there is no consensus in the assessment of mitochondrial function. In this review, the effects of polyphenols, ω-3 fatty acids and dietary fiber on mitochondrial function included studies that assessed mitochondrial function by changes in mRNA levels of mitochondrial markers, alterations in mitochondrial protein levels, enzymatic activity of key components of mitochondria-driven oxidation, substrate oxidation, as well as changes in mitochondrial size and shape. Although, since there is not a unique biomarker of its function, contradictory results are observed in the literature.

## 3. Polyphenols and Mitochondria

Polyphenols are plant-derived compounds with pleiotropic biological activities. Currently, over five hundred different polyphenols have been described in regularly-consumed foods. Although they exhibit far more activities than antioxidant ones, they are most widely known as antioxidants and therefore protector biomolecules against oxidative damage.

As mitochondria are the major cellular source of ROS, redox-active compounds can be targeted to those organelles to modulate the levels of ROS and the processes they induce. However, as antioxidants, it should be clear if the mechanism of action is derived by a direct interaction with oxidizing species (redox potential) or by indirect pathways that may modulate metabolism or mitochondrial functioning, therefore regulating the redox environment.

In the first case, cellular uptake of polyphenols is expected, and the rate of uptake will determine their biological activity. However, the rate of polyphenols’ uptake *in vivo* is dependent on the cell type, intracellular metabolism and rate of export [16]. Gut and hepatocyte uptake *in vivo* could be demonstrated by the fact that polyphenol metabolites are found in plasma after their oral intake. *In vitro* studies have also observed cellular uptake of flavonoids in T-cell lymphocytes, the mitochondria being the principal reservoir compared to other cellular compartments [17]. In red blood cells, it was described that quercetin is rapidly and avidly taken via a passive diffusion mechanism, driven by flavonoid binding to hemoglobin and resulting in an almost quantitative accumulation of the flavonoid [18]. In addition, in primary cultures of rat cerebellar granule neurons, epigallocatechin-3-gallate, a major flavonoid component of green tea, 90%–95% of the polyphenol was found accumulated in the mitochondrial fraction [19]. Nevertheless, the *in vivo* uptake of flavonoids in adipose tissue, muscle and brain is not so clear. Moreover, on the basis of the discussion below, large differences exist between the concentration of polyphenols required to exert an ROS-scavenging action *in vitro* and those likely to be attained after their dietary consumption. The actual possibility that polyphenols act *in vivo* through an ROS-scavenging mechanism has been increasingly questioned. Most *in vitro* studies indicate that the concentration of polyphenols required to efficiently scavenge ROS is generally between 10 and 100 µM. Such concentrations are greater than those reported to be reached in plasma after ingestion of a polyphenol-rich food. Therefore, it seems that the intracellular antioxidant action could be explained by a number of other mechanism, which generally require lower concentrations and do not involve the stoichiometric oxidative consumption of polyphenol molecules. In this regard, some polyphenols are able to induce the Keap1/Nrf2/ARE pathway for the transcription of cellular endogenous antioxidant defense. Compared to the classical direct ROS-scavenging antioxidant mechanism, the Keap1/Nrf2/ARE indirect mechanism of action will be more efficient, since it requires a lower concentration of polyphenols and does not depend on their direct stoichiometric consumption.

In the second case, without polyphenol cellular uptake, several studies have demonstrated the interaction of flavonoids with membrane receptors [20], as well as modifiers of cell membrane fluidity, microviscosity, order, elasticity and permeability [21], which may explain their biological effects in an indirect way.

Nevertheless, besides knowing the mechanism of action, it is clear that polyphenol intake may modulate metabolism and reverse a metabolic inflexibility condition. These compounds are now being recognized for their ability to modulate the capacity of mitochondria to undergo biogenesis; to control its membrane potential; and its ETC activity and ATP synthesis. A brief review of related studies is presented in Table 1.

In relation to mitochondrial biogenesis, resveratrol has been shown to be an effective inductor of SIRT1-mediated deacetylation of PGC-1α, activating its transcriptional activity in mice liver and muscle; which resulted in an increased number of mitochondria in the studied tissues [22]. Similarly, quercetin is able to induce the expression and activation of SIRT1 and PGC-1α and to increase the content of mtDNA and cytochrome c in both skeletal muscle and brain [23]. In humans, the administration of epicatechin-rich cocoa to patients with type 2 diabetes and heart failure, a stimulation in mitochondrial biogenesis in skeletal muscle, as expressed by an increment in SIRT1-dependent activation of PGC-1α and as a higher content of porin and mitochondrial complexes I and V, was observed [24].

In relation to the ETC, in aged rats, wine polyphenols totally restored muscle maximal mitochondrial oxidative capacity, normalizing ROS production in association with enhanced antioxidant defenses [31]. Coumestrol, found in a variety of legumes, increased the mitochondrial content in a dose-dependent manner in cultured skeletal muscle cells [25]. Moreover, the protein expression of ETC components, such as NDUFA9, SDHA, UQCRC2, COX1 and ATP5a, and transcriptional regulators that are responsible for mitochondrial biogenesis, such as PGC-1α and NRF-1, were augmented with coumestrol stimulation. Anthocyanidins, water-soluble pigments of colored berries, fruits and vegetables, can alleviate mitochondrial respiratory dysfunction caused by inhibition of complex I in human and rat tissues [26,27]. In isolated mitochondria from isquemic hearts, anthocyanins recovered complex I activity, therefore resulting in an increase in State 3 respiration and in the rate of ATP synthesis [28]. In this case, the suggested mechanism of action was attributed to its redox potential, where anthocyanins can function as electron carriers in a similar way as endogenous coenzyme Q1. Some polyphenols have also been reported to be able to induce mitochondrial uncoupling and/or to modulate the mitochondrial permeability transition pore (MPTP). In a study using isolated rat liver mitochondria, at 25–50 μM, the flavonol galangin was able to reduce the ∆Ψ_m_ and stimulate State 4 respiration [29]. The uncoupling effect of the flavonoids has been attributed to their weak acidic and overall high lipophilic nature, which is consistent with their putative ability to be protonated in the low-pH external side of the inner mitochondrial membrane, to pass through the lipid layer and to be de-protonated in the high-pH mitochondrial matrix milieu, thus dissipating the proton gradient across the inner mitochondrial membrane [32]. Beyond what exactly are the mechanisms by which polyphenols induce oxidative phosphorylation uncoupling, several investigators coincide in that such effects should be associated with a diminished rate of ROS formation through the ETC [33].

Our experience in polyphenols’ effects in insulin resistance suggests that the metabolic effect of its supplementation will depend greatly on the type of polyphenols. For example, the effects of epigallocatechin gallate in energy metabolism may be related to its ability to modulate energy uptake leading to mitochondrial adaptations [30]. Within this experiment, it was observed that the energy uptake by both gut and different peripheral tissues was reduced, where an increase in AMPKα was observed and further modifications in mitochondrial architecture; suggesting that the main effect of this type of polyphenol was a calorie-restriction mimicking one. In the opposite way, soy phytoestrogenic polyphenols may modulate energy metabolism by the stimulation of estrogen receptor pathways. Increased levels of estrogen may promote nuclear transcription of NRF-1 and further an increase in the transcription of nuclear genes that encode mitochondrial proteins involved in oxidative phosphorylation [34]. Additionally, it has been observed that soy isoflavones may improve insulin signaling [35], increasing cellular energy uptake capacity. However, at this point, a warning should be raised, since although in both cases of polyphenol treatments (epigallocatechin gallate and isoflavones), clinical parameters of insulin resistance and obesity will be improved, and long-term treatment will lead to different end-points. The reduction of energy uptake will lead to a lower energy production and, therefore, a reduced oxidative stress status. In the opposite way, an increase in energy uptake and metabolization will lead to an increase in energy production with the concomitant increase in oxidative stress. Unpublished observations from our laboratory demonstrate that although soy isoflavones improve insulin resistance parameters in a high-fat diet, an increase in general protein oxidation was observed, while the effect of green tea supplementation was a reduction in general protein oxidation.

## 4. ω-3 Fatty Acids and Mitochondria

Naturally-occurring ω-3 long-chain polyunsaturated fatty acids, namely eicosapentaenoic acid (EPA; 20:5n-3) and docosahexaenoic acid (DHA, 22:6n-3), are now regarded as healthy constituents of diets for diabetic patients. The metabolic effects of ω-3 fatty acids primarily result from their interactions with several organ systems. In the liver, hypolipidemic effects are observed due to a decrease in lipogenesis, a lower formation of triacylglycerols and a lower release of very low density lipoproteins into circulation, as well as an increase in fatty acid oxidation [36]. In skeletal muscle, the insulin-sensitizing effect of ω-3 fatty acids could be due to the increased glucose uptake and utilization [37]. For example, rats fed with ω-3 fatty acids utilize less oxygen for a given twitch force [38], suggesting that the improvement of mitochondrial efficiency could be either from a greater content of the electron transfer complexes or from enhanced kinetics of existing proteins. ω-3 fatty acids probably induce a switch in substrate metabolism, namely by an increase in fat oxidation and a decrease in carbohydrate oxidation [39,40]. They also affect the development of adipose tissue, as well as its metabolism and secretory functions [41]. A brief review of studies that observed effects in ω-3 fatty acids’ supplementation in mitochondria and/or metabolism is presented in Table 2.

Growing evidence suggest that dietary ω-3 fatty acids have a profound effect on mitochondria membrane phospholipid composition and, therefore, mitochondria function. Similar to cell membranes, mitochondrial membranes are rich in phosphatidylethanolamine (PE) and phosphatidylcholine (PC). However, unlike other membranes in mammalian cells, mitochondrial membranes contain high levels of cardiolipin, a tetra-acyl phospholipid. Cardiolipin comprises 10%–20% of the mass of total mitochondrial phospholipids, and its depletion results in severe mitochondrial dysfunction. Cardiolipin is necessary for the formation of contact sites between inner and outer mitochondrial membranes, stabilization of essential inner membrane proteins and respiratory complexes and in mitochondrial apoptotic signaling pathways [42]. Linoleic acid is the main fatty acyl moiety in cardiolipin, with 60%–80% of cardiolipin being tetralinoleoyl cardiolipin. Possibly, the mechanism of action of ω-3 fatty acids could be explained by the fact that changing membrane dietary fatty acid composition may modify the thickness, stiffness and fluidity of the lipid bilayer. It has been suggested that DHA is required for the organization and function of membrane proteins. This may be important in mitochondria, where electron transfer is tightly coupled between the complexes of the embedded electron transport chain. Furthermore, it has been observed that the unsaturation index of the mitochondrial membrane in rodents is positively associated with rates of palmitate oxidation. However, no changes in ω-3 incorporation into cardiolipin have been observed, although there was a strong trend for reductions in both linoleic and arachidonic acid content in cardiolipin following ω-3 supplementation [43].

In humans, ω-3 fatty acids supplementation (3 g for 12 weeks) induced an 8.8- and 3.9-fold increase in mitochondrial PC and PE fractions, respectively, while DHA incorporation increased 6.4- and 2.9-fold in PC and PE fractions, respectively, while also increasing 2.9-fold in the phosphatidylserine fraction [43]. However, no differences were found in mitochondrial respiration capacity and kinetics, and it does not alter the OXPHOS protein content. This could be explained, in part, because despite the observed changes in membrane composition, no differences in membrane microviscosity and fluidity were observed [44]. In this study, the change in ω-3 fatty acids was at the expense of ω-6 fatty acids without modifying the content of saturated fatty acids, even in a high saturated fat diet, explaining the lack of observed effects in membrane fluidity. 

Mitochondrial phospholipid remodeling with ω-3 fatty acids is further associated with delayed Ca^2+^-induced MPTP opening. Mitochondria determine cell survival through the opening of MPTP, which occurs under conditions of cell stress, causing mitochondrial depolarization and triggering of cell death. It was found that dietary supplementation with a mixture of DHA + EPA (70:30) increased the tolerance of isolated mitochondria to Ca^2+^-induced MPTP opening [45], with a decrease in hydrogen peroxide production [46]. The mechanism for greater resistance to Ca^2+^-induced mitochondrial permeability transition following ω-3 fatty acids supplementation is not clear. Although, it is suggested that an increase in DHA membrane content may slow Ca^2+^ uptake in response to an extra-mitochondrial upload, since its supplementation did not affect mitochondrial Ca^2+^ content, but attenuated the acute increase in mitochondrial Ca^2+^ following norepinephrine stimulation in isolated mitochondria from normal rat hearts [47]. This notwithstanding, it is necessary to mention that the enrichment of the mitochondrial membrane with polyunsaturated fatty acids bears the risk of lipid peroxidation. For instance, it has been described that DHA hydroperoxides apparently induced apoptosis and a dose-dependent attenuation of the mitochondrial potential [48].

The apparent discrepancy between reported findings may be explained by the possibility that ω-3 fatty acids may enhance mitochondrial function only under conditions where dysfunction is evident. Fish oil supplementation, for example, enhanced the respiratory control ratio and significantly improved the ATP levels in mitochondria from the brains of aged mice [49], where adult control mice had a significantly reduced activity of the respiration system complexes I, II and IV. These last authors suggested that fish oil improved mitochondrial function by modulating the expression of mitochondria-related genes. In *in vitro* hepatic cell culture, EPA and DHA induced an increase in Mfn2 mRNA levels, where an increase in the length of mitochondrial tubes was observed, increasing ATP and reducing ROS levels [50]. In another possible mechanism of action, comparing young with old mice, 10 weeks of EPA supplementation partially restored skeletal muscle mitochondrial capacity by increasing the coupling efficiency of the mitochondrial electron transport chain [51]. In this study, although no differences were found on mitochondrial biogenesis or abundance, a reduction in protein oxidative carbamylation was observed. Since protein carbamylation is catalyzed by myeloperoxidases, which is most abundant in neutrophils and has been found within skeletal muscle under conditions of neutrophil infiltration in response to inflammation, the possible anti-inflammatory effects of EPA may be driving improvements in mitochondrial function. 

Finally, the effects on mitochondrial function are reflected in the flexibility of the metabolism. Prior to metabolic inflexibility, in obesity, fatty acid metabolism is accelerated with possible accumulation of incomplete oxidation products, which may exacerbate insulin resistance. The accumulation of short-chain and long-chain acylcarnitines reflects a metabolic block of β-oxidation, when fatty acids are metabolized only partially. In this sense, ω-3 supplementation improves β-oxidation efficiency, where a reduction of even side-chain (C > 10) acylcarnitines, aroused in obesity controls, was significantly reduced. Additionally, several genes involved in carbohydrate metabolism showed significant changes in expression, such as pyruvate dehydrogenase kinase 4, a regulatory enzyme limiting oxidation of glucose by inhibiting the pyruvate dehydrogenase complex; fructose-1,6-biphosphatase 2, a key enzyme of gluconeogenesis catalyzing the hydrolysis of fructose 1,6-biphosphatase to fructose 6-phosphate; and glucose transporter 4, which is essential for the insulin-stimulated glucose uptake in muscle cells. In regards to lipid metabolism, genes encoding acyl-CoA thioesterase, a mitochondrial enzyme hydrolyzing medium and long-chain acyl-CoA to free fatty acids and CoASH, carnitine palmitoyltransferase 1b, the rate-limiting transporter of activated fatty acids for mitochondrial β-oxidation in the muscle, and CD36 protein, acting as a plasma membrane inducible transporter, were regulated by ω-3 fatty acids [52].

## 5. Dietary Fiber, Gut Microbiota and Derived Colonic Fermentation Metabolites and Mitochondria

Dietary fiber is defined as all food matrix components resistant to the action of digestive enzymes and, therefore, are not absorbed in the small intestine and could serve as substrates for fermentation by the colonic microbiota. Epidemiological evidence suggests that populations who follow a fiber-rich, traditional diet are likely to have a lower body weight and improved metabolic parameters than their Western-diet counterparts. Dietary fiber could play a role in the management of metabolic syndrome through its ability to control body weight evolution through its effect on satiety; to modulate glucose homeostasis/insulin sensitivity and to positively affect factors implicated in cardiovascular diseases. Recent experimental data suggest that the modification of gut peptides (involved in appetite and glucose homeostasis) could constitute a ‘metabolic relay’, allowing specific dietary fiber to act on appetite and other components of metabolic syndrome [53]. The efficacy of dietary fiber differs according to its dietary sources (fruits, legumes or cereals), but also to its specific chemical structure, responsible for its physical properties (*i.e.*, gel-forming capacity) or for its fermentation capacity in the lower part of the gut. 

The plausible mechanism of action of fiber on mitochondrial function could be derived by its ability to reduce energy intake by the inhibition of intestinal absorption of nutrients, the neuronal and satiety effects induced by its fermentation and bulking effect, the colonic fermentation by-products, mainly short-chain fatty acids (SCFA), that could serve as substrates for ATP synthesis and by the type of microbiota and microbiota metabolism. 

The first evidence about the role of gut microbiota on the development of obesity came from studies conducted on germ-free mice (GF-mice) compared to conventionally-raised mice [54]. In basal conditions, the latter have a 40% higher body fat content than GF-mice, and this phenomenon was independent of food intake. In a subsequent study, it was observed that GF-mice, fed with a “high sugar-high-fat Western diet”, do not seem to develop obesity. The main mechanisms explaining GF resistance to diet-induced obesity are the enhanced fatty acids oxidation, uncoupled with decreased lipoprotein lipase activity and fatty acids storage [55]. The lean phenotype was associated with increased skeletal muscle and liver levels of phosphorylated AMPK and its downstream targets involved in fatty acid oxidation (acetyl-CoA carboxylase; carnitine-palmitoyltransferase). Other studies have reported specific changes of gut microbiota composition in genetically obese mice, compared to lean counterparts, showing a 50% reduction in the abundance of *Bacteroidetes* and a proportional increase in *Firmicutes* [56]. These specific changes could contribute to the increased SCFA production and energy harvest [57]. From another point of view, it is suggested that gut microbial ecology is to a large extent modulated by diet in humans; a high intake of fat and proteins is associated with increased levels of *Bacteroides*, whereas high fiber intake is associated with increased levels of *Prevotella* [58,59]. Recently, metagenomic analysis showed that the gut microbiota of responders of a dietary fiber treatment was enriched in *Prevotella copri* and had increased potential to ferment complex polysaccharides [60]. In the same study, the microbiota enriched in *Prevotella* was transplanted to germ-free mice in a high-fat diet; compared to non-responder microbiota, the *Prevotella-*transplanted mice contained higher amounts of glycogen in the liver. However, it is not clear how and why, in obese subjects, gut microbiota seems to extract more energy from ingested food. To identify metabolic pathways associated with obesity, metabolic reconstructions of the “core” microbiome revealed significant enrichment in phosphotransferase systems involved in microbial processing of carbohydrates and in genes involved in carbohydrate, lipid and amino acid metabolism [61]. Therefore, except for the cases of bacterial translocation, the main effects of fiber intake in metabolic inflexibility may be derived from the actions of colonic fermentation products.

The main colonic bacterial fermentation products of dietary fiber are SCFAs, such as acetate, propionate and butyrate. SCFAs can be used for *de novo* synthesis of lipids and glucose, which are the main energy sources for the host. SCFAs are good candidates for explaining the biological effects of fiber consumption in metabolic syndrome, since their bioavailability is almost complete, and they can serve as substrates for obtaining energy in the mitochondria.

Recently, two orphan G-protein-coupled receptors (GPCR), GPR41 and GPR43, were reported to be activated by SCFAs. For example, in adipose tissue, SCFA-mediated GPR43 activation suppressed adipose insulin signaling, leading to inhibition of fat accumulation, and unincorporated lipids and glucose were primarily utilized in muscles where the expression of energy expenditure, glycolysis and β-oxidation-related genes was increased [62]. In another study, the incorporation of sodium butyrate (5% *wt*/*wt* in a high-fat diet) induced higher energy expenditure and oxygen consumption during the night in mice. The calorie intake was also higher without a difference in body weight compared to the control group, suggesting an increase in fatty acid oxidation that was confirmed by monitoring ^14^C-labeled palmitic acid, and a 200% increase in ^14^C-labeled CO_2_ in butyrate-treated mice was observed. In the same study, an obesity treatment experiment was performed, where after five weeks of treatment with butyrate, the obese mice lost 10.2% of their body weight, reduced their fasting glucose by 30% and their HOMA-IR by 50%. The authors explain the observed effects by PGC-1α activation properties in brown fat, skeletal muscle and liver [63].

## 6. Conclusions

There is ample evidence of the positive effects of several classes of polyphenols, ω-3 fatty acids and dietary fiber on the reversion of a metabolic inflexibility condition. Although the mechanism of action is not well establish, in some cases, an indirect interaction with mitochondrial metabolism is expected, where most of the described studies suggest the activation of NRF-1, AMPKα, SIRT or PGC-1α as the main mechanism of action. In the case of polyphenols, due to their reduced bioavailability, a direct interaction with mitochondrial metabolism is not expected. However, indirect activation of pathways involved in energy metabolism may induce function and morphological changes in mitochondria. ω-3 fatty acids have been demonstrated to be directly interacting with mitochondria, since their incorporation in the phospholipids of the mitochondrial membrane is observed. However, their incorporation does not induce changes in mitochondrial activity, suggesting that the mechanism of action could be the indirect stimulation of fatty acids’ β-oxidation, possibly by the activation of PPAR transcriptional factors. Dietary fiber is a bioactive compound for which it is more difficult to explain its mechanism of action, since it is not absorbed due to its molecular weight, and several fermentation by-products could be produced during its fermentation in the colon, each one with pleiotropic effects in metabolism. The most plausible mechanism of action could be due to its energy restriction properties and the production of SCFAs, which may induce fatty acids’ β-oxidation. In all cases, it should be taken into account that the main problem in metabolic inflexibility is the accumulation of energy due to its high intake, and the main treatment objective should be addressed toward a reduction in energy intake. The use of bioactive compounds, such as the ones included in this review, could facilitate the treatment, and they should not be considered as complete problem-solving agents.

## Figures and Tables

**Table 1:** Described effects of dietary phenolic compounds in mitochondria and metabolic flexibility.

Compound	Effect	Mechanism	Type of Study	Reference
Resveratrol	Increased number of mitochondria in liver and muscle	SIRT1 and PGC-1α activation	Animal model	[22]
Quercetin	Increased mtDNA and cytochrome *c* content in muscle and brain	SIRT1 and PGC-1α activation	Animal model	[23]
Epicatechin-rich cocoa	Mitochondrial biogenesis stimulation in muscle	SIRT1 and PGC-1α activation	Human study	[24]
Coumestrol	Increased mitochondrial content in muscle cells	SIRT1 activation	Cell culture	[25]
Quercetin, kaempferol, epicatechin	Inhibitors of H_2_O_2_ production by mitochondria	Inhibition of complex I activity	Cell culture	[26]
Grape seed proanthocyanidin extract	Enhanced thermogenic capacity and improvement in mitochondrial function in brown and adipose tissue	Not described	Animal model	[27]
Anthocyanins	Complex I activity recovery and increase in the rate of ATP synthesis	Functioning as electron carriers in a similar way as coenzyme Q1	Isolated mitochondria	[28]
Galangin	Modulation of the mitochondrial permeability transition pore	Decreased fluidity of the mitochondrial membrane	Isolated mitochondria	[29]
Epigallocatechin	Modification in mitochondrial architecture	AMPKα activation	Animal model	[30]

**Table 2:** Described effects of dietary phenolic compounds in mitochondria and metabolic flexibility.

Product	Effect	Mechanism	Type of Study	Reference
Fish oil	Improvement in mitochondrial efficiency	Increased content or enhanced kinetics of ETC	Animal model	[38]
Fish oil	Reduced body fat mass	Stimulation of lipid oxidation	Human study	[39]
Fish oil	Decrease in insulinemia	Increased lipid oxidation	Human study	[40]
DHA + EPA	Improve in mitochondrial ADP kinetics	Incorporation in mitochondrial membranes, displacing ω-6 species in several phospholipids population	Human study	[44]
DHA + EPA	Decrease in H_2_O_2_ production	Increased tolerance to Ca^2+^-induced MPTP opening	Isolated mitochondria	[47]
Fish oil	Improvement in ATP production in brain	Improvement in membrane fluidity	Animal model	[49]
EPA and DHA	Increase in ATP and reduction in ROS levels in hepatocytes	Increase in the length of mitochondrial tubes by an increase in Mfn2 mRNA levels	Cell culture	[50]
EPA	Restoration of skeletal muscle mitochondrial capacity	Increase in coupling efficiency of the ETC	Animal model	[51]
